# Crosstalk between Photoreceptor and Sugar Signaling Modulates Floral Signal Transduction

**DOI:** 10.3389/fphys.2017.00382

**Published:** 2017-06-12

**Authors:** Ianis G. Matsoukas

**Affiliations:** School of Life Sciences, University of WarwickCoventry, United Kingdom

**Keywords:** cryptochromes, developmental phase transitions, floral signal transduction, florigen, juvenile-to-adult phase transition, photoreceptors, phytochromes, sugar signaling

## Abstract

Over the past decade, integrated genetic, cellular, proteomic and genomic approaches have begun to unravel the surprisingly crosstalk between photoreceptors and sugar signaling in regulation of floral signal transduction. Although a number of physiological factors in the pathway have been identified, the molecular genetic interactions of some components are less well understood. The further elucidation of the crosstalk mechanisms between photoreceptors and sugar signaling will certainly contribute to our better understanding of the developmental circuitry that controls floral signal transduction. This article summarizes our current knowledge of this crosstalk, which has not received much attention, and suggests possible directions for future research.

## Introduction: light, sugars and floral signal transduction

Post-embryonic development progresses through distinct developmental phase transitions. It has been proposed (Matsoukas, 2014a) that the prolonged juvenile-to-adult and vegetative-to-reproductive phase transitions might be due to several antiflorigenic signals, which affect the transcription levels of florigen *FLOWERING LOCUS T* (*FT*; Corbesier et al., 2007), and *SQUAMOSA PROMOTER BINDING PROTEIN-LIKE* (*SPL*; Shikata et al., 2009) genes.

Juvenility can be defined as the early period of development during which the abundance of antiflorigenic signals such as miR156/miR157 (Lauter et al., 2005; Martin et al., 2009; Lee et al., 2010; Varkonyi-Gasic et al., 2010) is sufficiently high to suppress the expression of *FT* and *SPLs* (Shikata et al., 2009, 2012; Wang et al., 2009; Jung et al., 2011). On the other hand, expression of miR172 in leaves activates *FT* (Aukerman and Sakai, 2003; Jung et al., 2007), through repression of AP2-like transcripts *SCHLAFMÜTZE* (*SMZ*), *SCHNARCHZAPFEN* (*SNZ*) and *TARGET OF EAT 1–3* (*TOE1-3*; Jung et al., 2007; Mathieu et al., 2009), whereas the increase in *SPL*s at the shoot apical meristem (SAM), leads to the activation of floral meristem identity genes (Wang et al., 2009; Yamaguchi et al., 2009), which result in vegetative-to-reproductive phase transition.

Light is a key regulator of the juvenile-to-adult and vegetative-to-reproductive phase transitions (Turck et al., 2008; Matsoukas et al., 2012; Lifschitz et al., 2014; Matsoukas, 2015). It constitutes a critical environmental growth indicator, which is estimated by the duration, quality, direction and intensity, as well as the essential energy source for the synthesis of carbohydrates by the photosynthetic apparatus. Light perception is mediated through the action of photoreceptors, namely PHYTOCHROMES (PHYs; derives from Greek phyto- “relating to plants” and khrōma “color”; Chen and Chory, 2011), CRYPTOCHROMES (CRYs; derives from Greek kruptós “hidden” and khrōma “color”; Chaves et al., 2011), the ultraviolet B photoreceptor ULTRA VIOLET RESISTANCE LOCUS 8 (Jenkins, 2014), phototropins (Christie, 2007) and the ZEITLUPE (ZTL) family members ZTL, FLAVIN-BINDING, KELCH-REPEAT F-BOX (FKF1), and LOV KELCH PROTEIN 2 (LKP2; Kim et al., 2007; Suetsugu and Wada, 2013). Members of each of these photoreceptor families have direct interactions with circadian clock genes and proteins.

Several molecular mechanisms that mediate sugar responses have been identified in plants (reviewed in Rolland et al., 2006; Smeekens et al., 2010; Dobrenel et al., 2013; Lastdrager et al., 2014; Smeekens and Hellmann, 2014; Van den Ende, 2014; Li and Sheen, 2016). Sugar signals can be generated either by carbohydrate concentration and relative ratios to other metabolites, such as hormones and carbon-nitrogen ratio, or by flux through sugar-specific transporters and/or sensors (Matsoukas, 2014b). Glucose, sucrose and trehalose-6-phosphate (T6P) have been recognized as pivotal integrating regulatory molecules that control the expression of genes involved in floral signal transduction (reviewed in Ponnu et al., 2011; Bolouri Moghaddam and Van den Ende, 2013; Matsoukas, 2014b).

Glucose-mediated signal transduction is largely dependent on HEXOKINASE1 (HXK1)-dependent pathway, HXK1-independent pathway, and glycolysis-dependent pathway, which utilizes the SUCROSE NONFERMENTING RELATED KINASE1 (SnRK1)/TARGET OF RAPAMYCIN (TOR) pathway (Moore et al., 2003; Baena-Gonzalez et al., 2007; Ren et al., 2012). SnRK1 has a role when sugars are in extremely limited supply, whereas HXK and Tre6P play a role in the presence of excess sugar. Sucrose plays an essential role in the regulation of important metabolic processes (reviewed in Tognetti et al., 2013). Its concentration tends to be directly related to light intensity (LI), and inversely related to temperature. It has been shown that sucrose, together with T6P act as proxies for the carbohydrate status in plant tissues (Lunn et al., 2006; Wahl et al., 2013; Xing et al., 2015). It is notable that T6P inhibits the activity of the SnRK1 in sugar metabolic control of floral signal transduction (Zhang et al., 2009). In particular, mutations in *SNRK1* confer early flowering, whereas *SnRK1* overexpression delays flowering (Baena-Gonzalez et al., 2007; Tsai and Gazzarrini, 2012). Several lines of evidence suggest that Tre6P inhibits SnRK1 when sucrose is above a threshold level (Polge and Thomas, 2007; Zhang et al., 2009). When the sucrose concentration decreases, with Tre6P decreasing as well, SnRK1 is released from repression, promoting the expression of genes involved in photosynthesis-related events, so that more carbon is made available (Delatte et al., 2011). Mutations in T6P signaling pathway confer late flowering. This late flowering phenotype was found to be due to reduced expression levels of FT, the elevated levels of miR156, and reduced levels of at least three miR156-regulated transcripts: *SPL3, 4, 5* (Wahl et al., 2013). However, T6P not only signals sucrose availability (Lunn et al., 2006), but it also negatively regulates sucrose levels by restricting sucrose synthesis and/or promoting sucrose catabolism (Yadav et al., 2014). Interestingly, the regulatory effects of T6P on growth and development would be an effective means for manipulating carbon partitioning and plant yield (Smeekens, 2015).

The identification of downstream components of photoreceptor signaling that involved in floral signal transduction has revealed a crosstalk between pathways of different light qualities as well as with other seemingly unrelated signaling pathways. One such crosstalk that has not received much attention and involves carbohydrates, forms the focus of this article.

## Light perception and circadian clock

The circadian [derived from the Latin roots “*circa*” (around) and “*diem*” (day)] system is a complex regulatory network. It is consists of a set of proteins that forms an interconnected feedback network with multiple loops. This system provides temporal information to organisms to coordinate developmental and metabolic responses in coincidence with the environmental conditions. One of the main functions of light in regulation of floral signal transduction is in the initiation of cues that interact with the circadian oscillator and entrain the circadian rhythm. Several reviews have been published on the circadian clock system recently (Romanowski and Yanovsky, 2015; Endo, 2016; Sanchez and Kay, 2016), so the circadian clock will not be described in great detail here. The circadian clock system has three primary components. First is the central oscillator/pacemaker that generates the 24 h oscillators. A model for the *Arabidopsis* circadian oscillator described a series of multiple interlocked transcriptional–translational feedback loops referred to as the morning, core, and evening loops (Huang et al., 2012; Pokhilko et al., 2012). The “morning complex” comprises the genes encoding the proteins CIRCADIAN CLOCK ASSOCIATED 1 (CCA1; Wang and Tobin, 1998) and LATE ELONGATED HYPOCOTYL (LHY). Both genes increase their expression prior to dawn (Schaffer et al., 1998). The “morning complex” genes encoding PSEUDO-RESPONSE REGULATOR (PRR) 5, 7, and 9 increase their expression after dawn (Matsushika et al., 2000; Farre et al., 2005). The “evening loop” comprises genes encoding GI (Fowler et al., 1999; Park et al., 1999) and TIME OF CAB EXPRESSION 1 (TOC1; Strayer et al., 2000) as well as the evening complex genes encoding EARLY FLOWERING (ELF) 3, 4 (Herrero et al., 2012), and LUX ARRHYTHMO (LUX; Hazen et al., 2005; Nusinow et al., 2011). The “evening complex” genes increase their expression prior to, and after dusk. The “morning” and “evening” complex proteins regulate each other through a series of promoter *cis*-acting elements (Harmer et al., 2000; Alabadi et al., 2001; Covington et al., 2008), and protein–protein interactions (Kim et al., 2007; Nusinow et al., 2011; Chow and Kay, 2013). These type of interactions create a robust and tunable oscillator that modulate gene expression in a coordinated 24 h rhythm.

The second component is the input pathway that synchronizes or entrains the oscillator with environmental cues. The best-characterized signal is light (reviewed in Kami et al., 2010). In *Arabidopsis*, red/far-red light perception is mediated by PHYs. Blue light perception is mediated by CRYs and the blue-light sensing proteins ZTL, FKF1, and LKP2. The third component is the output pathway that links the oscillator to processes under circadian rhythm such as photoperiodic induction and floral signal transduction.

The plant circadian oscillator is also entrained by daily temperature rhythms (Wenden et al., 2011) and sugars (Blasing et al., 2005; Dodd et al., 2005; Knight et al., 2008; Dalchau et al., 2011; Haydon et al., 2013). However, the perception and transduction of such signals are not fully understood. Considering that photosynthates can contribute to the fine-tuning of the circadian clock (reviewed in Sanchez and Kay, 2016) and that floral signal transduction in LDs is also controlled by the circadian clock (Matsoukas et al., 2012; Song et al., 2013), it has been hypothesized that photosynthates might have a role in modulating the photoperiodic timing mechanism, which includes the PHYs and CRYs (Dodd et al., 2015).

PRRs have been identified as components of the circadian clock (Nakamichi et al., 2007; Ito et al., 2008). Generally, it has been proposed that PRRs contribute to photoperiod measurement through regulation of the time-keeping mechanism associated with CO transcription (Strayer et al., 2000; Yanovsky and Kay, 2002; Nakamichi et al., 2007, 2010). Recently, it was shown that PRRs form a light-signaling mechanism dedicated to photoperiodic flowering through their accumulation during the day, transferring information on light exposure to CO protein (Hayama et al., 2017), which acts upstream of FT and TSF. Interestingly, PRR7 expression is coordinately modulated not only by light but also by photosynthesis, permitting PRR7 to act as a transcriptional repressor in circadian sugar signaling (Haydon et al., 2013). Therefore, specific circadian-clock components not only transfer temporal information to a photoperiodic time-keeping mechanism but also convey qualitative and quantitative information on light exposure to the time-keeping mechanism, establishing measurement of day length.

## Interplay between sugar and phytochrome signaling modulates floral signal transduction

In *Arabidopsis*, the *PHY* family consists of *PHYA, PHYB, PHYD*, and *PHYE* (Table 1; Clack et al., 1994). *PHYA* is predominately involved in physiological responses to continuous far-red light, whereas *PHYB* is involved in responses to red light. The *phyA* mutant flowers significantly later than wild type (WT) in long days (LDs), which indicates that *PHYA* acts to promote flowering (Johnson et al., 1994). In antithesis, the early flowering phenotype of *phyB* mutant under short day (SD) and LD conditions demonstrates the repressive role of *PHYB* in floral signal transduction (Guo et al., 1998). Interestingly, the identification of downstream components of photoreceptor-signaling that involved in floral induction has revealed a crosstalk between pathways of different light qualities as well as with other seemingly unrelated pathways such as phytohormones (Matsoukas, 2014b) and carbohydrate metabolism-related events (Dijkwel et al., 1997; Short, 1999; Kozuka et al., 2005; Ghassemian et al., 2006).

**Table 1:** List of genes that are discussed in this mini review.

**Gene name**	**Abbreviation**	**Allelic**	**Gene identifier**	**Description**	**References**
*ABA INSENSITIVE4*	*ABI4*	*ATABI4; GIN6; ISI3; SALOBRENO 5; SAN5; SIS5; SUN6; T7M7.16*	AT2G40220	*ABI4* involved in ABA signal transduction, ABA-mediated glucose response, and HXK-dependent sugar responses.	Finkelstein et al., 1998; Arenas-Huertero et al., 2000
*CHLOROPHYLL A/B-BINDING PROTEIN*	*CAB*	*AB165; F1N18.4; F1N18_4; LHCB1.1*	AT1G29920	Encodes lhcb1.1, a component of the LHCIIb light harvesting complex associated with photosystem II.	Friso et al., 2004; Cottage and Gray, 2011
*CHLOROPLASTIC β-AMYLASE3*	*BAM3*	*AtBAM3; BAM3; BETA-AMYLASE 3; BMY8; DL4575C; FCAALL.5*	AT4G17090	*BAM3* encodes a β-amylase targeted to the chloroplast.	Lao et al., 1999; Mccallum et al., 2000; Kaplan and Guy, 2005
*CRYPTOCHROME-INTERACTING BASIC-HELIX-LOOP-HELIX 1*	*CIB1*	*T4L20.110; T4L20_110*	AT4G34530	CIB1 acts together with additional CIB1-related proteins to promote *CRY2*-dependent floral signal transduction. CIB1 promotes florigen expression.	Liu et al., 2008
*CONSTANS*	*CO*	*B-BOX DOMAIN PROTEIN 1; BBX1; F14F8.220; F14F8_220; FG*	AT5G15840	CO promotes floral signal transduction in response to LDs, is modulated by the circadian clock and day length.	Wenkel et al., 2006
*CRYPTOCHROME1*	*CRY1*	*ATCRY1; BLU1; HY4; OOP2; OUT OF PHASE 2; T3H13.14; T3H13_14*	AT4G08920	CRY1 functions in perception of blue / green ratio of light.	Valverde et al., 2004
*CRYPTOCHROME2*	*CRY2*	*AT-PHH1; ATCRY2; F19P19.14; F19P19_14; FHA; PHH1*	AT1G04400	Blue light receptor. It is a positive regulator of floral signal transduction via *CO*.	Ahmad et al., 1995
*FLAVIN-BINDING, KELCH REPEAT, F BOX 1*	*FKF1*	*ADO3; F BOX 1; T23K23.10*	AT1G68050	FKF1 forms a complex with GI on the *CO* promoter to regulate the expression of *CO*.	Nelson et al., 2000
*FLOWERING LOCUS T*	*FT*	*F5I14.3; F5I14_3; REDUCED STEM BRANCHING 8; RSB8*	AT1G65480	FT protein is the long-sought florigen, or at least, part of it.	Kardailsky et al., 1999; Kobayashi et al., 1999; Corbesier et al., 2007
*GIGANTEA*	*GI*	FB; T22J18.6; T22J18_6	AT1G22770	GI promotes floral signal transduction under LDs, in a circadian clock-controlled floral induction pathway. Starch excess mutant.	Eimert et al., 1995; Tseng et al., 2004; Penfield and Hall, 2009
*ELONGATED HYPOCOTYL 5*	*HY5*	*F2I11.150; F2I11_150; TED 5*	AT5G11260	HY5 is a central mediator of CRY and PHY responses.	Lee et al., 2007
*LOV KELCH PROTEIN 2*	*LKP2*	*ADAGIO 2; ADO2*	AT2G18915	Overexpression of LKP2 results in arrhythmic phenotypes, and a loss of photoperiodic control of floral signal transduction.	Schultz et al., 2001
*microRNA156a*	*miR156a*	*Ath-MIR156a;* gene family: *MIPF0000008;* Accession: *MI0000178*	Next upstream gene: At2g25090; next downstream gene: At2g25100	*Arabidopsis* miR156 is an ambient temperature-responsive miRNA. It plays an important role in regulating floral signal transduction.	Telfer et al., 1997; Telfer and Poethig, 1998; Aukerman and Sakai, 2003; Wu and Poethig, 2006
*microRNA157b*	*miR157b*	*Ath-MIR157b;* gene family: *MIPF0000008;* Accession: *MI0000185*	Next upstream gene: At1g66790; next downstream gene: At1g66800	Overexpression of *Arabidopsis* miR157b induces bushy architecture and delayed juvenile-to-adult phase transition	Shikata et al., 2012; May et al., 2013
*microRNA172a*	*miR172a*	*Ath-MIR172a;* gene family: *MIPF0000035;* Accession: *MI0000215*	Next upstream gene: At2g28050; next downstream gene: At2g28060	miR172 mediates light signals from GI and promotes floral signal transduction in *Arabidopsis* by inducing *FT*.	Jung et al., 2007; Wu et al., 2009
*PHYTOCHROME A*	*PHYA*	*ELONGATED HYPOCOTYL 8; F14J9.23; F14J9_23; FHY2; FRE1; HY8*	AT1G09570	Light-labile cytoplasmic red/far-red light photoreceptor involved in floral signal transduction.	Whitelam et al., 1993; Reed et al., 1994
*PHYTOCHROME B*	*PHYB*	*HY3; MSF3.17; MSF3_17; OOP1; OUT OF PHASE 1*	AT2G18790	PHYB regulates the expression of genes in response to red light. It repress floral signal trusnduction.	Koornneef et al., 1980; Reed et al., 1994
*PHYTOCHROME D*	*PHYD*	*DL4165C; FCAALL.323*	AT4G16250	Encodes a phytochrome photoreceptor with a function similar to that of *PHYB*.	Reed et al., 1994
*PHYTOCHROME E*	*PHYE*	*F15J5.100; F15J5_100*	AT4G18130	PHYE is member of Histidine Kinase. Mutation in *PHYE* confers early flowering.	Reed et al., 1994; Devlin et al., 1998
*PLASTOCYANIN*	*PETE 1*	*T23E18.3; T23E18_3*	AT1G76100	One of two *Arabidopsis* plastocyanin genes. *PETE1* is essential for electron transport.	Abdel-Ghany, 2009; Pesaresi et al., 2009
*PLASTOCYANIN*	*PETE 2*	*DRT112; F14O10.6; F14O10_6;*	AT1G20340	One of two *Arabidopsis* plastocyanin genes. It is expressed 10x higher than *PETE1*.	Abdel-Ghany, 2009; Pesaresi et al., 2009
*RIBULOSE 1,5-BISPHOSPHATE CARBOXYLASE/OXYGENASE*	*RBCS*	*OSRBCS; RBCS-C; OsJ_016909*	LOC4351966	Encodes a member of the Rubisco small subunit multigene family in *Oryza sativa*.	Takano et al., 2009
*SCHLAFMÜTZE*	*SMZ*	*T15C9.6*	AT3G54990	Encodes an AP2 domain transcription factor that can repress floral signal transduction.	Mathieu et al., 2009
*SCHNARCHZAPFEN*	*SNZ*	*T16B24.11; T16B24_11*	AT2G39250	Encodes an AP2 domain transcription factor that can repress floral signal transduction.	Mathieu et al., 2009
*SQUAMOSA PROMOTER BINDING PROTEIN-LIKE 3*	*SPL3*	*T1B8.11; T1B8_11*	AT2G33810	*SPL3* is involved in regulation of floral signal transduction. Its temporal expression is regulated by miR156.	Jung et al., 2011; Wahl et al., 2013
*SQUAMOSA PROMOTER BINDING PROTEIN-LIKE 4*	*SPL4*	*F8L10.12; F8L10_12; FTM6;*	AT1G53160	*SPL4* is involved in regulation of floral signal transduction. Its temporal expression is regulated by miR156.	Jung et al., 2011; Wahl et al., 2013
*SQUAMOSA PROMOTER BINDING PROTEIN-LIKE 5*	*SPL5*	*n/a*	AT3G15270	*SPL5* is involved in regulation of floral signal transduction. Its temporal expression is regulated by miR156.	Jung et al., 2011; Wahl et al., 2013
*SQUAMOSA PROMOTER BINDING PROTEIN-LIKE 13A*	*SPL13A*	*MBA10.13; MBA10_13; SPL13*	AT5G50570	*SPL* genes function in distinct pathways to promote different adult vegetative phase traits and floral induction. *SPL13A* and *SPL13B* encode the same protein.	Cardon et al., 1999; Xing et al., 2010
*SQUAMOSA PROMOTER BINDING PROTEIN-LIKE 13B*	*SPL13B*	*MFB16.6; SPL13*	AT5G50670	S*PL13B* and *SPL13A* encode the same protein.	Cardon et al., 1999; Xing et al., 2010
*SQUAMOSAPROMOTER BINDING PROTEIN-LIKE 15*	*SPL15*	*n/a*	AT3G57920	Encodes a transcriptional regulator that is involved in the vegetative-to-reproductive phase transition. Its expression is regulated by miR156b.	Cardon et al., 1999; Schwarz et al., 2008
*SQUAMOSAPROMOTER BINDING PROTEIN-LIKE 9*	*SPL 9*	*AtSPL9; T24P15.11; T24P15_11*	AT2G42200	Encodes a putative transcriptional regulator that is involved in the vegetative to reproductive phase transition. Expression is regulated by miR156b.	Cardon et al., 1999; Schwarz et al., 2008; Wang et al., 2008; Xing et al., 2010
*SUCROSE-PHOSPHATE SYNTHASE*	*SPS*	*ATSPS1F, SPS1F, SPSA1, SUCROSE-PHOSPHATE SYNTHASE A1*	AT5G20280	Encodes a protein with putative sucrose-phosphate synthase activity.	Park et al., 2008
*SUCROSE TRANSPORTER 4*	*SUT4*	*ATSUC4; ATSUT4; F21M12.35; F21M12_35; SUC4*	AT1G09960	*AtSUT4* is expressed in companion cells contributing, along with *AtSUC2*, to phloem loading.	Schulze et al., 2003
*SUCROSE UNCOUPLED 6*	*SUN6*	*ATABI4; GIN6; ISI3; SAN5; SIS5; T7M7.16*	AT2G40220	Involved in ABA signal transduction, ABA-mediated glucose response, and HXK-dependent sugar responses.	Arenas-Huertero et al., 2000
*SWEET11*	*SWEET11*	*NODULIN MTN3 FAMILY PROTEIN; AtSWEET11; T21J18.1*	AT3G48740	Encodes a member of the SWEET sucrose efflux transporter family proteins.	Chen et al., 2012
*SWEET12*	*SWEET12*	*BIDIRECTIONAL SUGAR TRANSPORTER SWEET12-LIKE PROTEIN*	AT5G23660	Encodes a member of the SWEET sucrose efflux transporter family proteins.	Chen et al., 2012
*TARGET OF EARLY ACTIVATION TAGGED EAT 1*	*TOE1*	*RAP2.7; T17D12.11; T17D12_11*	AT2G28550	TOE1 is member of the AP2 family. AP2 regulates floral signal transduction through regulating *SOC1* and *FT* expression.	Aukerman and Sakai, 2003; Jung et al., 2007; Mathieu et al., 2009; Yant et al., 2010; Zhang et al., 2015
*TARGET OF EARLY ACTIVATION TAGGED EAT 2*	*TOE2*	*MGO3.10; MGO3_10*	AT5G60120	TOE2 is member of the AP2 family. Overexpression of *TOE*s confer late flowering.	Aukerman and Sakai, 2003; Jung et al., 2007; Mathieu et al., 2009; Yant et al., 2010; Zhang et al., 2015
*TARGET OF EARLY ACTIVATION TAGGED EAT 3*	*TOE3*	*K21H1.22; K21H1_22*	AT5G67180	TOE3 is member of the AP2 family.	Aukerman and Sakai, 2003; Jung et al., 2007; Mathieu et al., 2009; Yant et al., 2010; Zhang et al., 2015
*TREHALOSE-6-PHOSPHATE SYNTHASE*	*TPS1*	*ATTPS1; T30F21.9; T30F21_9*	AT1G78580	*TPS1* synthesizes T6P. Knockdown of *TPS1* reduces T6P cellular concentrations and represses floral signal transduction.	Van Dijken et al., 2004; Wahl et al., 2013
*TWIN SISTER OF FT*	*TSF*	*F9F13.20; F9F13_20*	AT4G20370	*TSF* Encodes a floral inducer that is a homolog of FT. Mutant lines overexpressing *TSF* flower earlier than WT.	Yamaguchi et al., 2005
*ULTRA VIOLET RESISTANCE LOCUS 8*	*UVR8*	*MGI19.7; MGI19_7*	AT5G63860	UV-B-specific signaling component that orchestrates expression of a range of genes with vital UV-protective functions.	Rizzini et al., 2011
*ZEITLUPE*	*ZTL*	*ADO1; FKF1-LIKE PROTEIN 2; FKL2; LKP1; MSF19.2; MSF19_2*	AT5G57360	The protein contains a PAS domain ZTL that contributes to the plant fitness carbon fixation, biomass by regulating the circadian clock.	Somers et al., 2000

Carbohydrates modulate development through PHY-mediated responses (Tsukaya et al., 1991; Barnes et al., 1996; Dijkwel et al., 1997; Short, 1999). *PHYA* is involved in activation of several photosynthetic genes, such as *RIBULOSE 1,5-BISPHOSPHATE CARBOXYLASE/OXYGENASE* (*RBCS), CHLOROPHYLL A/B-BINDING PROTEIN* (*CAB*), and *PLASTOCYANIN* (*PC*). *CAB, RBCS*, and *PC* are repressed by sucrose or glucose (Dijkwel et al., 1997; Takano et al., 2009; Cottage and Gray, 2011). Exogenous sucrose application or high light intensity (LI) reverses the late-flowering phenotype of the *Arabidopsis phyA* mutant. It has been proposed that the late-flowering phenotype of *phyA* might be due to a reduced photosynthetic input to *FT* (King et al., 2008). This is supported by the fact that high LI reverses its late flowering phenotype, the mutant has half the WT leaf area and, in addition, a reduced photosynthetic pigment content (Walters et al., 1999; Bagnall and King, 2001; King et al., 2008).

Overexpression of *PHYs* in *Nicotiana tabacum* (Sharkey et al., 1991) and *Solanum tuberosum* (Sharkey et al., 1991; Yanovsky et al., 1998) increase the transcription of *SUCROSE-PHOSPHATE SYNTHASE* (*SPS)*. Interestingly, ectopic expression of *SPS* has been shown to promote flowering in several plant species (Micallef et al., 1995; Baxter et al., 2003). On the other hand, loss of *PHYs* in *Oryza sativa phyA phyB phyC* triple mutant affect sugar metabolism, carbon partitioning and sugar transport (Jumtee et al., 2009). In *Arabidopsis*, the circadian regulated sugar-induced β*-AMYLASE3* (*BAM3*) gene is induced by *PHYA* transcription (reviewed in Kaplan et al., 2006). *BAM3* is essential for maltose production (Niittyla et al., 2004), whereas it regulates the juvenile-to-adult and vegetative-to-reproductive phase transitions via starch catabolism-related events (Matsoukas et al., 2013).

The *SUCROSE UNCOUPLED6 (SUN6)* gene of *Arabidopsis* is involved in hexose kinase-mediated sugar sensing (Huijser et al., 2000). Gene expression analysis in the sugar insensitive *sun6* mutant has shown that *PHYA* signaling is not repressed by sugars (Dijkwel et al., 1997). *SUN6* was shown to be allelic to *ABA INSENSITIVE 4* (*ABI4*). Functional analysis of the *abi4* mutant has shown that it is defective in ABA metabolism or response (Dijkwel et al., 1997; Huijser et al., 2000). Therefore, the early flowering phenotype of *sun6*, at least in LDs, demonstrates a tight interplay between light quality, sugar and phytohormone pathways in regulation of floral induction in *Arabidopsis*.

Further evidence on interaction between carbohydrate-metabolism repression and light signaling is provided by the inhibitory activity of *PHYB* in the control of hypocotyl elongation by *PHYA*, in presence of exogenous sucrose or glucose (Short, 1999). Down-regulation or over-expression of *SUT4* in *Solanum tuberosum* delays or promotes floral induction, respectively (Chincinska et al., 2008). Besides floral induction, in the same work evidence was provided on *SUT4* involvement in the shade avoidance response. This suggest that *PHY*-dependent and photoperiod-dependent developmental responses, such as floral signal transduction and shade avoidance share a common downstream mechanism in which sucrose accumulation levels are actively involved.

## Interplay between sugar and cryptochrome signaling modulates floral induction

CRYPTOCHROMES (CRYs) comprise flavoproteins that are able to detect blue light (Guo et al., 1998). The role of *CRY1* in promoting floral induction in *Arabidopsis* has been demonstrated by the late flowering phenotype of *cry1* mutants compared to WT in various light conditions (Mozley and Thomas, 1995). Similarly, the *cry2/fha1* (*fha-1* is a mutant allele of *CRY2* in Landsberg erecta background) mutant flowers later than the WT in LDs but not in SDs, whereas transgenic plants overexpressing *CRY2* flowered slightly early in SDs but not in LDs (Koornneef et al., 1991). It has been shown that *CRY2* interacts with bHLH proteins CRYPTOCHROME-INTERACTING BASIC-HELIX-LOOP-HELIX (CIB) proteins to regulate the *FT* expression and floral signal transduction (Liu et al., 2008; Liu H. et al., 2013; Liu Y. et al., 2013).

Further evidence for the interaction between photosynthetic assimilates and CRYs is provided by a microarray analysis revealing regulation of *CRY1* and *CRY2* transcription levels by glucose (Li et al., 2006). It has been reported that *PHYA* interacts with *CRY1*, and *PHYB* binds *CRY2* (Ahmad et al., 1998; Mas et al., 2000), so red and blue light may crosstalk at multiple layers to co-ordinately regulate developmental transitions. *PHYB, CONSTANS* (*CO)* and, indirectly, *PHYA* are under the regulation of *CRYs* (Valverde et al., 2004; Thomas, 2006). Therefore, any modification on *CRYs* transcription levels would also affect the other photoreceptors and *CO*, which act directly upstream of *FT* and *TWIN SISTER OF FT* (*TSF)* with catalytic effects on the juvenile-to-adult and vegetative-to-reproductive phase transitions.

Mutants lacking *CRY*s or having defects in their signaling pathway show changes in chloroplast composition and disturbance of normal acclimation (Smith et al., 1993; Walters et al., 1999). The fact that CRY1 and CRY2 can also act as sensors of irradiance (Guo et al., 1998) could provide a further link between light quality and carbohydrate metabolism in regulation of floral signal transduction.

The *Arabidopsis* ELONGATED HYPOCOTYL 5 protein (HY5) is a central mediator of CRY and PHY responses (Lee et al., 2007). It integrates multiple environmental and phytohormonal signaling inputs (Catala et al., 2011; Xu et al., 2014) by mediating homeostatic coordination of sugars (Chen et al., 2016), and maintaining chlorophyll levels and CO_2_ uptake. It appears that HY5 might operate in conjunction with the circadian oscillator to adjust levels of rhythmic photosynthetic gene expression (Toledo-Ortiz et al., 2014). Interestingly, HY5 regulates both sucrose metabolism and subsequent movement of sucrose into phloem cells for shoot-root translocation by promoting the expression levels of *SWEET11* and *SWEET12* (Chen et al., 2016), genes encoding sucrose efflux transporters (Chen et al., 2012), and *TPS1* (Chen et al., 2016), a gene encoding T6P. The T6P pathway controls the expression of SPLs, partially via miR156, and partly independently of the miR156-dependent pathway via the florigen *FT* (Wahl et al., 2013). Evidence have been provided that miR156, and possibly miR172, are directly regulated by HY5 (Zhang et al., 2011). Taken together, these data could provide a potential mechanistic link, at the molecular level, on how the photoreceptor-sugar crosstalk might be involved in regulation of floral signal transduction via the HY5 and *TPS1*-miR156-*SPL* module.

## Light intensity and floral signal transduction

**Figure 1:**
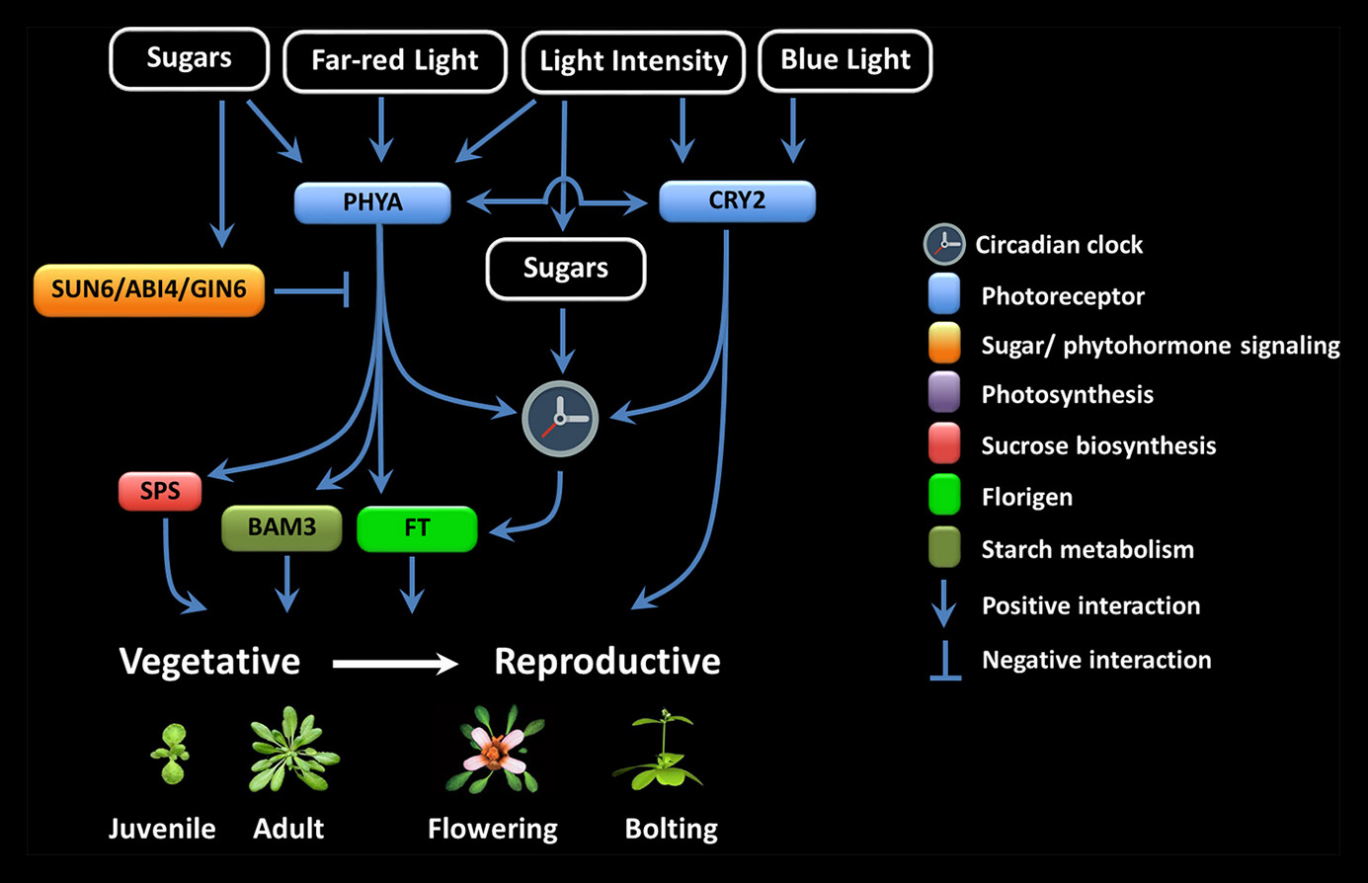
Multiple interactions among the components involved in floral signal transduction in response to photoreceptor and sugar signaling crosstalk.

LI seems to be particularly important during the juvenile-to-adult and vegetative-to-reproductive phase transition (Figure 1). It has been proposed that the inability to flower during the juvenile period is because of a foliar inability to produce floral signals, the presence of antiflorigens, and/or of the incompetence of the SAM to respond (Zeevaart, 1985; Matsoukas et al., 2012, 2013; Matsoukas, 2015). The length of the juvenile vegetative phase in daylenth-sensitive plants can be revealed by reciprocal transfers between inductive and non-inductive photoperiods (Adams et al., 2003; Matsoukas et al., 2013; Matsoukas, 2014a).

Exposure to low or high LI levels can delay or hasten time to flowering, respectively. For instance, *Achillea millefolium* grown under a 16 h d^−1^ photoperiod in controlled environment conditions flowered after 57, 45, and 37 d when grown under 100, 200, or 300 μmol m^−2^ s^−1^, respectively (Zhang et al., 1996). Similarly, Adams et al. (1999) demonstrated that Petunia flowering was hastened by LDs, but that decreased LI prolonged time to flowering. *Arabidopsis* plants flower rapidly under non-inductive SDs after exposure to 8–12 d at a high LI. It has been shown that this “photosynthetic” response is FT-independent. In contrast, the *IDD8* locus of *Arabidopsis* was reported to have a role in FT-dependent induction of flowering by modulating sugar transport and metabolism by regulating *SUCROSE SYNTHASE4* activity (Seo et al., 2011).

However, the effect of LI on time to flowering can be unpredictable in several species. Hence, the term “facultative irradiance response” (FI) has been coined to describe a developmental hastening of flowering by addition of supplemental light (Erwin and Warner, 2000). Species such as Antirrhinum [LD plant (LDP)], Nicotiana [LDP or SD plant (SDP)], and Petunia (LDP) that exhibit a FI response, show a decrease in leaf numbers and days to flower as irradiance increases. In contrast, the term “irradiance indifferent” (II) refers to species such as Salvia (SDP or facultative LDP) and Zinnia (day neutral plant or facultative SDP) that do not show any response to increased irradiance (Thomas and Vince-Prue, 1997; Erwin and Warner, 2000; Mattson and Erwin, 2005; Thomas, 2006).

Despite the high sensitivity of FI species to elevated levels of LI, the majority does not show a hastened flowering phenotype with increasing irradiance. It has been shown for *Pelargonium* x *hortorum* that a linear relationship between LI and days to flower, for an increased irradiance developmental response, exists until a threshold level between 6.89 and 9.01 μmol m^−2^ d^−1^ (Erickson et al., 1980). However, some species require greater threshold levels. For instance, absolute flowering of Digitalis was reached with LI > 11 μmol m d (Fausey et al., 2001). Furthermore, giving supplemental irradiance (at 30, 60, and 90 μmol m^−2^ s^−1^) to Gerbera hastened flowering by up to 23 d in the winter, but only up to 11 d during the Spring (Gagnon and Dansereau, 1989). This suggests that the impact of supplemental irradiance on floral signal transduction can be dependent on season's ambient light conditions and species' threshold requirement.

What is not clear is the precise molecular genetic mechanisms by which LI, if acting through photosynthates can regulate the floral signal transduction. It may well be that assimilates themselves act as part of the florigen (Périlleux and Bernier, 2002; Bernier and Perilleux, 2005). Interestingly, long-distance floral signal transport is now accepted as more complex than the movement of a single type of signal molecule (Matsoukas et al., 2012; Matsoukas, 2015). It is possible that total carbohydrate, or a particular carbohydrate level may be required to reach a specific threshold in order to sustain a steady supply of sufficient bulk flow through the phloem from the leaves to the SAM to enable delivery of florigen. This would be necessary to render the SAM competent to flower.

## Concluding remarks

Floral signal transduction has been the focus of a great deal of attention during the last few decades. The molecular mechanisms underlying light perception and the downstream signaling pathways that regulate the floral signal transduction have been intensively challenged. The fact that some photoreceptors can also act as sensors of irradiance provides a promising link between light qualities and assimilate partitioning and resource utilization in regulation of floral signal transduction.

Numerous reports highlight the role of several molecules that integrate light, clock, temperature, and hormone signaling pathways in orchestration of floral signal transduction. However, further investigation is vital for the elucidation of the molecular mechanism underlying photoreceptor-mediated signal integration at the subcellular, tissue-specific and temporal level in response to sugar signaling. This research field is prosperous and technical advances in “-OMICS” tools might shed light on the underlying molecular genetic mechanisms.

## Author contributions

The author confirms being the sole contributor of this work and approved it for publication.

### Conflict of interest statement

The author declares that the research was conducted in the absence of any commercial or financial relationships that could be construed as a potential conflict of interest.

## Nomenclature

The following nomenclature will be used in this article:

• Names of genes are written in italicized upper-case letters, e.g., *CRY2*.

• Names of proteins are written in non-italicized upper-case letters, e.g., CRY2.

• Names of mutants are written in italicized lower-case letters, e.g., *cry2*.
